# Effects of cannabinoids in Parkinson’s disease animal models: a systematic review and meta-analysis

**DOI:** 10.1136/bmjos-2022-100302

**Published:** 2022-12-19

**Authors:** Berzenn Urbi, Yunjoo Lee, Ian Hughes, Sarah Thorning, Simon A Broadley, Arman Sabet, Saman Heshmat

**Affiliations:** 1 Office for Research Governance and Development, Gold Coast University Hospital, Southport, Queensland, Australia; 2 Medicine, Griffith University Faculty of Health, Gold Coast, Queensland, Australia; 3 Department of Neurology, Gold Coast University Hospital, Southport, Queensland, Australia

**Keywords:** Cannabinoids, Receptors, Cannabinoid

## Abstract

**Objectives:**

Cannabis has been proposed as a potential treatment for Parkinson’s disease (PD) due to its neuroprotective benefits. However, there has been no rigorous review of preclinical studies to evaluate any potential treatment effect. This systematic review was undertaken to provide evidence in support or against a treatment effect of cannabinoids in animal models of PD.

**Methods:**

Databases were searched for any controlled comparative studies that assessed the effects of any cannabinoid, cannabinoid-based treatment or endocannabinoid transport blocker on behavioural symptoms in PD animal models.

**Results:**

A total of 41 studies were identified to have met the criteria for this review. 14 of these studies were included in meta-analyses of rotarod, pole and open field tests. Meta-analysis of rotarod tests showed a weighted mean difference of 31.63 s for cannabinoid-treated group compared with control. Meta-analysis of pole tests also showed a positive treatment effect, evidenced by a weighted mean difference of −1.51 s for cannabinoid treat group compared with control. However, meta-analysis of open field test demonstrated a standardised mean difference of only 0.36 indicating no benefit.

**Conclusion:**

This review demonstrates cannabinoid treatment effects in alleviating motor symptoms of PD animal models and supports the conduct of clinical trials of cannabis in PD population. However, there is no guarantee of successful clinical translation of this outcome because of the many variables that might have affected the results, such as the prevalent unclear and high risk of bias, the different study methods, PD animal models and cannabinoids used.

**STRENGTHS AND LIMITATIONS OF THIS STUDY**

- Our meta-analysis demonstrates a treatment effect of cannabinoids in improving motor symptoms of Parkinson’s disease (PD) animal model.
- Despite heterogeneity amongst included study and high risk of bias, our meta-analysis was aimed to detect a treatment effect of cannabinoids in PD animal model which it did.
- This systematic review justifies the conduct of investigating cannabis effect in patients with PD especially for motor functions.

## Introduction

Parkinson’s disease (PD) is the second most common neurodegenerative disorder.^1^ The median age of symptom onset is 60 years and the mean disease duration from diagnosis to death is 15 years^2^ with a mortality ratio estimated as 1.52:1 at 10 years in a meta-analysis of inception cohorts.^3^ The pathophysiology of PD is still not completely understood. However, the hallmark of the disease is the degeneration of dopamine producing neurons in the pars compacta of the substantia nigra.^4^ This results in the classic symptoms of rigidity, bradykinesia, tremor and postural instability.

Several genetic mutations have been identified in PD. However, the disease is predominantly a sporadic disorder in which 90% of cases are idiopathic.^1^ There are a number of human and animal studies supporting the role of oxidative stress and the inflammatory cascade in the development and progression of PD.^5^ The development of therapeutics that target these mechanisms has been proposed to benefit people with PD.

The endocannabinoid system regulates the physiological functions of the nervous system and may have neuroprotective functions. There have been arguments in support of cannabis as a potential treatment for patients with PD due to their antioxidative and anti-inflammation potential.^6 7^ However, studies of cannabis in patients with PD have been inconclusive.^8 9^ A systematic review of cannabinoids in PD animal models is worth investigating to better understand the different beneficial effects of cannabinoids and how it may assist in better designing of human clinical trials. This review aims to investigate preclinical evidence for treatment effect of cannabinoids in animal models of PD.

## Methods

### Search strategy

We searched Medline (Ovid), Embase (Elsevier), CINAHL (Ebsco) Cochrane Central Register of Controlled Trials, Proquest Dissertations and Thesis Global, Web of Science (Clarivate) and PsycINFO (Ebsco) on 22 July 2022. The search was conducted with no limits for date published, language or study type. The search strategy was developed in Medline using subject heading and keyword terms for Parkinson’s and Cannabis and translated for the other databases (refer to Table S1-S8) for full search strategy on https://doi.org/10.6084/m9.figshare.19695004.v3). No filter for finding animal studies or preclinical studies were used as this was part of a wider project on Cannabis and Parkinson’s Disease.

### Inclusion and exclusion criteria

We searched for any controlled comparative studies (randomised controlled trial (RCT), quasi-RCT and non-randomised) that assessed the effects of any cannabinoid, cannabinoid-based treatment or endocannabinoid transport blocker on behavioural symptoms in PD animal models, at any stage of the disease process, by any route, at any dose and for any duration. As such, the meta-analyses presented here aim to increase the probability of identifying the presence of any treatment effect rather than increasing the accuracy of an unknown but consistent treatment effect.

Studies that measured any behavioural PD symptoms were included in this review. This included motor symptoms of PD assessed by objective motor observations such as rotarod, pole and open field tests, and non-motor symptoms of PD assessed by neuropsychological evaluation and hyposmia evaluation in rodent models.^10 11^

Rotarod and pole tests are behavioural motor assessments used specially to measure bradykinesia in 1-Methyl-4-phenyl-1,2,3,6-tetrahydropyridine (MPTP)-treated rodents.^12^ The rotarod test measures the time the animal remains on a rotating rod while the pole test measures the time it took for an animal to turn downward and descend from the top of the pole to the base (or cage).^10^ A longer time spent on the rotating rod and the shorter time descending the pole conveys better motor functions in the animal. The open field test, on the other hand, measures locomotor activity by recording free movement of the animal on a horizontal plane in a monitored cage or chamber and is usually expressed in the overall distance travelled though no standard durations or field areas have been established.^11^ A longer distance travelled conveys better locomotor function of the animal.

Studies that used PD animal models and met the required criteria for the following categories were included in this review: toxin-based, transgenic or pesticide/herbicide models.^13 14^

Studies that investigated the effects of cannabinoids in vitro or lacked behavioural assays were excluded. Narrative reviews, letters, editorials, case reports or those without objective data available to be evaluated, were also not included. The protocol for this review was published in PROSPERO (CRD42019157162). There were no major changes or deviations from the protocol in completing this review. Also, we had emailed some of the authors included in this review to request raw data or details of any unpublished and incomplete trials but did not get any response. Preferred Reporting Items for Systematic Reviews and Meta-Analyses (PRISMA) reporting guideline was used in this review.^15^

### Study selection

Two reviewers (YL and BU) independently screened the titles and abstracts identified from the literature search using the protocol published in PROSPERO. Full texts of all potentially relevant studies were downloaded for independent assessment and data extraction by the two reviewers. Similar papers published in different forms (ie, conference abstract and full article) were grouped together for review. Papers with more completed data (eg, full article instead of abstract) were included in this review. Any disagreement with the article selection was resolved through discussion with a third reviewer (IH).

### Data extraction

Quantitative data from the included articles were extracted using the data extraction and assessment template from the Cochrane Public Health Group.^16^ Means, sample sizes and SEs were extracted from papers and included careful measurement of column heights and error bars on enlarged figures. Details on the intervention, animal model, study methods and outcomes of significance were extracted. In some studies, multiarm treatments were used (ie, different dosing and/or cannabinoid types). Meta-analysis assumes studies are independent, hence, for this review, only one treatment-control comparison was included. The following criteria were used to determine which treatment group was included in this meta-analysis. For studies with multiarm dosing, the highest dosing group was chosen for analysis. For studies with multiple cannabinoid-type arms, the most used cannabinoid among all other included studies was chosen for analysis to minimise heterogeneity.

### Study quality

The quality of each included article was addressed using the criteria in the SYRCLE’s risk of bias (RoB) tool for conducting systematic reviews in experimental non-human studies.^17^ Studies were assessed against the following biases: selection bias (sequence generation, baseline characteristics, allocation concealment), performance bias (random housing, blinding), detection bias (random outcome assessment, blinding), attrition bias (incomplete outcome data), reporting bias (selective outcome reporting) and other (other sources of bias).

### Statistical analysis

**Figure 1:**
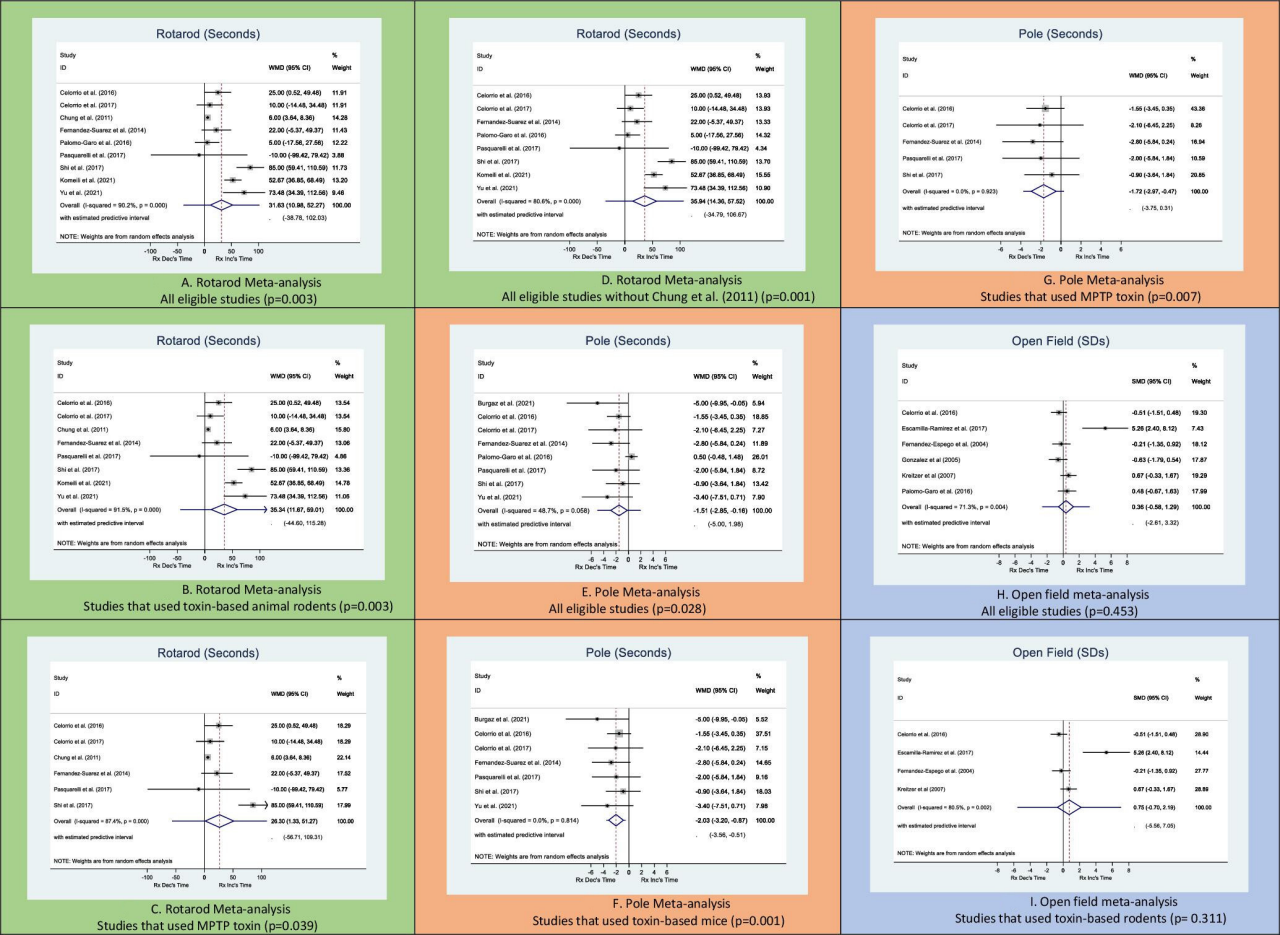
Meta-analyses for rotarod, pole and open field tests.

Stata V.17 packages metan, metaan and mvmeta were used to perform random effects meta-analyses. Presented results were obtained using the DerSimonian and Laird method. Meta-analyses of rotarod and pole tests were performed using weighted mean differences (WMD) while meta-analysis of open field tests used standardised mean differences (SMDs) due to the lack of consistency in field dimensions or test duration. Summary means are presented with their 95% CIs. All studies for rotarod performed three tests per mouse and the mean time from the three trials was used to calculate an overall mean for each of the treatment and control groups. Consequently, the SEs of the means presented for these studies are comparatively smaller than the SEs from the single trial studies. To adjust for this, so that the three trial studies could be combined with the single trial studies, the conversion SE_1_=SE_3_ $\sqrt{3}$ was used where the subscript refers to the number of trials. $\sqrt{n}$), where *n* is the sample size of the treatment or control group. Heterogeneity, or the degree of between study differences, was measured using the *I^2^* statistic, which describes the percentage of total variation across studies that is due to heterogeneity. Forest plots (figure 1) and funnel plots (Fig S4) on https://doi.org/10.6084/m9.figshare.19695004.v3)^18^ are presented. Sensitivity analyses were performed in which certain studies were excluded due to a potentially influential difference in methodology. For example, Pasquarelli *et al*^19^ used an oral route of administration while other studies used an intraperitoneal (i.p.) route and^20 20^ used a transgenic animal model whereas other studies used toxins to induce the animal analogue of PD.

## Results

### Design of studies

Electronic and hand searching resulted in 3079 potential articles with 66 of these were found to be eligible for full texts screening. Of these, 42 met all criteria and were included in this review. One conference abstract^21^ was replaced in the review by its full article^22^ making it 41 articles included. Figure S1 (https://doi.org/10.6084/m9.figshare.19695004.v3)^18^ presents the PRISMA flow diagram of the search, screening and selection process of studies. The reasons for excluding each article during the full-text review are also presented.

Of the 41 studies included in this review, 2 studies^20 23^ used transgenic animal models (ie, Parkin (Park-2) and leucine-rich repeat serine/threonine kinase knock out mice), 3 studies used MPTP-treated marmosets,^24–26^ 1 study used MPTP-treated drosophila^27^ while the rest of the 41 studies used MPTP or 6-hydroxydopamin (6-OHDA)-treated rodents (mice or rats).

Cannabinoid interventions from included studies varied from cannabinoid receptor (CBR) agonists such as HU308, CBD, WIN55,212–2, HU210, nabilone, THC, CP55,940, ACEA, CBGA-quinone (CBGA-Q), GW842166x and AM1241,^20 23 24 26–52^ endocannabinoid enzyme inhibitors (FAAH and MAGL) which increase available endocannabinoids such as JZL184, URB597, KML29, PF-3945,^19 21 22 25 29 34 45 53–56^ a putative endocannabinoid, noladin ether which is an endocannabinoid agonist,^32 57 58^ an endocannabinoid modulator blocking anandamide reuptake called AM404^59^ preserves the supply of endocannabinoids and beta-caryophellene, which is a terpene commonly found in cannabis.^60^

There were 14 studies included in 3 meta-analyses (green highlights on Table S9) https://doi.org/10.6084/m9.figshare.19695004.v3).^18^ One study^51^ was initially considered for rotarod meta-analysis however the study used percentage of baseline as a measure rather than seconds, which prevented its inclusion in meta-analysis (orange highlight on Table S9) https://doi.org/10.6084/m9.figshare.19695004.v3).^18^ Seven studies measured both rotarod and pole tests.^19 20 22 30 41 50 53^ Two additional studies measured rotarod^31 47^ only, giving a total of nine studies included in the meta-analysis. There was also one additional study^45^ that assessed pole test only giving eight studies in the meta-analysis. Another six studies presented data on open field test.^20 23 34 53 54 59^ One of these six studies^20^ performed both rotarod and pole tests which was also included in those meta-analyses while the other five studies were not.

Of the nine studies in the rotarod meta-analysis, eight used toxin-based rodent models of PD^19 22 30 31 41 47 50 53^ while seven of the eight studies in pole meta-analysis used toxin-based rodent models.^19 22 30 41 45 50 53^ One study (included in both rotarod and pole test groups)^20^ used a transgenic PD animal model. Subgroup analyses of studies that used only toxin (MPTP or 6-OHDA)-based rodents in rotarod and pole tests were undertaken as well as studies that used only MPTP-based rodents.

Of the six studies included in the open field meta-analysis, four used toxin-based animal models: two 6-OHDA-treated rodents^54 59^ and two MPTP-treated mice.^34 53^ The four studies that used toxin-based animal models were also separately meta-analysed.

Detailed characteristics of all included studies for this review are presented in Table S9, which can be accessed on https://doi.org/10.6084/m9.figshare.19695004.v3.^18^

### Reported study quality

The overall quality of 42 included studies was assessed as mostly unclear and high RoB (Fig S2-S3), (Table S10) on https://doi.org/10.6084/m9.figshare.19695004.v3).^18^ There is uncertainty from included studies in determining whether different biases were addressed. This is compounded by the lack of international guidelines in conducting PD preclinical studies causing variabilities on the study design, methods of performing different tests, consideration of proper randomisation techniques, behavioural assessments and the use of blinding.

### Meta-analysis of rotarod

Nine studies^19 20 22 30 31 41 47 50 53^ performed rotarod tests and were considered for meta-analysis. Cannabinoid interventions used varied: URB597, an FAAH inhibitor,^53^ JZL184 and KML29, both MAGL inhibitors,^19 22^ and CBD, HU308, HU210, THC, GW842166x and AM1241, all CB agonists.^20 30 31 41 47 50^ Treatment periods ranged from 28 to 60 days,^19 22 30 53^ except for three studies where treatment periods were either 180 days^20^ or between 12 and 26 days.^41 47 50^ Interventions were administered i.p. except for one study,^19^ which was administered orally. All but one study^31^ administered the cannabinoid-based treatment ranging from 8 hours to 1 week after the toxin induction. Sensitivity analyses were undertaken based on treatment timing, and genetic or toxin-based models—weighted and SMDs did not significantly change the result (figure 1A–D)

Of the total nine studies for rotarod test, a random effects model was used in the meta-analysis. A WMD of 31.627 s (95% CI 10.98 to 52.27 s; p=0.003) on the rotating rod was seen for cannabinoid treated compared with control PD animal models (figure 1A). Heterogeneity was high (I^2^=90.2%) (figure 1A) indicating considerable heterogeneity.

We then meta-analysed studies that used only toxin-based animal models, that is, MPTP-treated or 6-OHDA-treated rodents, producing an outcome from a less heterogeneous animal group of nine studies. Only one study used a transgenic PD animal model.^20^ The remaining articles used either MTPTP (n=6) or 6-OHDA (n=2) toxins in rodents. A WMD of 35.34 s (95% CI 11.67 to 59.01; p=0.003) on the rotating rod was seen for cannabinoid-treated mice compared with control (figure 1B). Again, heterogeneity was high (I^2^=91.5%) (figure 1B) indicating considerable heterogeneity.

We further investigated whether there was a treatment effect when just assessing the MPTP-based studies. A WMD of 26.30 s (95% CI 1.33 to 51.27; p=0.039) on the rotating rod was seen for cannabinoid-treated mice compared with control (figure 1C). Heterogeneity was slightly lower but still high ((I^2^=87.4%).

Lastly, we excluded Chung *et al*^31^ as it included treatment both before and after toxin induction. A WMD of 35.94 s (95% CI 14.36, 57.52; p=0.001) on the rotating rod for cannabinoid-treated mice compared with control.

### Meta-analysis of pole test

Eight studies presented data on pole tests.^19 20 22 30 41 45 50 53^ Cannabinoids used in each study were: URB597 and KML-29, FAAH inhibitors,^19 53^ JZL184 a MAGL inhibitor,^22^ CBR agonists CBD, HU-308, GW842166x and AM1241^20 30 41 47 50^ and a cannabinoid precursor, CBGA-Q.^45^ All studies administered treatments ranging from 8 to 24 hours after the toxin delivery. Treatment periods for most studies lasted between 35 and 60 days, with three studies^41 45 50^ having treatment periods lasting between 12 and 26 days. Treatments were administered i.p. except for one^19^ where the oral route was used. A WMD of −1.51 s (95% CI −2.85 to –0.16; p=0.028) descending time from pole was seen for cannabinoid-treated mice compared with control (figure 1E). The eight studies included in pole meta-analysis had low heterogeneity (I^2^=48.7%) (figure 1E).

Seven of the eight studies used toxin-based C57BL/6 PD mice while the other study used transgenic PD mice.^20^ The seven studies that used toxin-based PD mice were meta-analysed. Two studies used 6-OHDA-treated C57BL/6 mice^19 50^ while the remaining five studies used MPTP-treated C57BL/6 mice.^22 30 41 45 53^ Cannabinoid-treated C57BL/6 mice produced a WMD of −2.03 s in descending time (95% CI −3.20 to –0.87; p=0.001) compared with control (figure 1F). Heterogeneity of these seven studies is very low (*I^2^*=0.0%).

Meta-analysis of studies that used MPTP-based mice was undertaken. Five studies included in this subgroup analysis. Cannabinoid-treated mice produced a WMD of −1.72 s in descending time (95% CI −2.97 to –0.47; p=0.007) (figure 1G).

### Meta-analysis of open field

Six studies^20 23 34 53 54 59^ presented data on the open field test. Studies differed on how distance travelled on the open field was measured and the duration of observation being 5 min,^20 23 53^ 10 min^34 59^ or 15 min.^54^ There were also variations in the cannabis intervention used in each study: AM404, an endocannabinoid modulator,^59^ THC and HU-308, both CB agonists^20 23^ and URB597, an FAAH inhibitor.^34 53 54^ Treatment duration ranged from 5 days to 6 months, and all studies delivered treatment ranging from 2 hours to 1 month after toxin induction.

Due to how distance was measured in each study, meta-analysis was conducted using the SMD. An SMD of 0.36 (95% CI −0.58 to 1.29; p=0.453) was observed between cannabinoid treated and control rodents (figure 1H). Of these six studies, four used toxin-based animal models.^34 53 54 59^ These were meta-analysed with an SMD of 0.75 (95% CI −0.70 to 2.19; p=0.311) between cannabinoid treated and control rodents (figure 1I).

Heterogeneity was high (I^2^=71.3%) (figure 1H) likely due to the different measurement protocols used for open field in each study.

For each meta-analysis, small study bias was assessed by visual inspection of funnel plots.^18^ Asymmetry in the rotarod and pole funnel plots suggest potential small study bias but also reinforce the observation of heterogeneity between studies. Distinct groups of methodologically dissimilar studies may be the prime driver of the observed asymmetry.

### Effects of cannabinoids on PD non-motor symptoms

Three studies assessed non-motor PD symptoms. One study evaluated the effect of a FAAH inhibitor, URB597, on psychosis in MPTP lesioned marmosets-treated with L-DOPA.^25^ Psychosis was operatively defined as hyperkinesia, response to nonapparent stimuli (representing hallucinatory behaviour), repetitive grooming and stereotypies. Scores omposed of cumulative counts of recorded behaviours. Although, L-DOPA treatment increased psychosis in PD-induced marmosets, URB597 had no measurable effect on psychosis compared with vehicle. URB597 had a selective reduction in hyperkinesia but not psychosis overall. The other two studies investigated learning and cognitive function by measuring the step-down test.^49 56^ Both studies used toxin PD animal models, either MPTP^49^ or 6-OHDA.^56^ The mice were trained to learn to step down from the platform in order to avoid an electrical shock prior to administering the toxin. One study found that URB597 (p<0.001) had a significant effect of retaining learnt memory compared with the control PD group.^56^ Likewise, the second study showed that CBD and L-dopa independently and significantly increased avoidance times of the shock, representing learnt behaviour, compared with the control PD group.^49^

## Discussion

This systematic review demonstrates potential efficacy of cannabinoids in improving motor functions in toxin-based rodent models of PD measured by both rotarod and pole behavioural tests. The meta-analyses provide evidence justifying the conduct of clinical trials investigating cannabis or cannabis-based treatment of PD in humans, particularly for studies intending to show an improvement of motor function as their primary focus. However, this does not guarantee successful translation clinically. Included studies have high or unclear risk of bias, which might have affected the outcomes. To investigate whether cannabis has any potential benefit for patients with PD, a properly designed RCT is needed.

A meta-analysis of rotarod results from nine studies demonstrated an estimated treatment effect of 31.63 s (95% CI 10.98 to 52.27 s; p=0.003) increased retention time on the rotating rod in favour of the cannabinoid-treated group. For studies that used only toxin-based rodent models, there was increased retention time on the rotating rod of 35.34 s (95% CI 11.67 to 59.01; p=0.003) and for studies that specifically used MPTP toxin PD rodents, 26.30 s (95% CI 1.33 to 51.27; p=0.039). A reduction in descending time from the pole was also demonstrated in the meta-analysis with an estimated treatment effect of −1.51 s (95% CI −2.85 to –0.16; p=0.028). For studies that only used toxin-based rodents, an estimated treatment effect of −2.03 s in descending time (95% CI −3.20 to –0.87; p=0.001) was observed while for studies that used MPTP toxin only had an estimated treatment effect of −1.72 s in descending time (95% CI −2.97 to –0.47; p=0.007) in favour of cannabinoids.

Rotarod and pole tests are common and reliable objective behaviour assays used in PD animal experiments.^13 14^ Both measurements are used to quantify progress or improvement in bradykinesia and postural stability, common symptoms in PD.^61^ The rotarod test requires animals to stay on the rod that is rotating faster than normal walking speed, and to have sufficient dopamine and reticulospinal tract function^12^ to execute this activity. Pole test involves forelimb grasping and manoeuvring to turn and climb down from the top of the pole, which requires intact basal ganglia and sufficient rubrospinal tract function.^12^ Although these two measurements are reliable and often used in PD animal experiments, there are identified limitations.

Both tests require animal models to be pretrained prior to the actual testing.^62^ Most of the studies in this meta-analysis described pretraining of their animal models. Additionally, as with any animal testing, there are always external factors that create stress to animals being tested, which may influence their overall performance and behaviour.^63 64^ For instance, mice are naturally social animals and follow social hierarchy. A male mouse may dominate other mice in the same cage by guarding food supply thereby limiting intake in other mice.^65 66^ Given the variability in animal testing environments, there are factors possibly impacting the results of behavioural examinations: the timing, dosage and total duration of interventions administered, the degree of dopamine loss in the disease course and when the behavioural assays were measured. Some mice were considered reverse cycled (nocturnal)^67^ but tested during daylight. Despite these limitations, there is no single behaviour assay in rodents that captures all the motor deficits of human PD. The rotarod and pole tests are the more common and reliable tests we have readily available for preclinical testing in PD.

The toxin rodent PD models are appropriate to use when investigating neuroprotective interventions such as the case with cannabinoids because of the high replicability of PD motor symptoms in this category of animal models.^62^ However, there are also known limitations with neurotoxic animal models such as absence of typical PD intraneural proteinaceous Lewy’s bodies, particularly in MPTP-treated or 6-OHDA-treated rodents.^62^ Toxin-based PD rodents are currently one of the most widely accepted experimental models that has been invaluable in better understanding PD mechanisms and in screening potential treatments. In fact, PD medications such as amantadine, a glutamate antagonist widely used for dyskinesia, were successfully translated to clinical use because of experiments conducted using MPTP-treated animal models. Deep brain stimulation for advanced PD is another treatment that was pioneered on toxin-based rodent models.^62^

There are also known differences of PD animal models even within the toxin-based category. Toxins used for PD animal models such as reserpine produce parkinsonian symptoms, however, their effects are temporary and do not lead to dopamine (DA) neuronal death.^68^ A better yet imperfect alternative is the 6-OHDA toxin which is more reliable in damaging DA neurons and producing parkinsonism. However, its effects are acute and does not produce complete composite symptoms of PD. This toxin is also often injected unilaterally rather than bilaterally into the substantia nigra, the nigrostriatal tract of the striatum. Bilaterally lesioned animal models often die from marked aphagia, adipsia and seizures.^69^ This outcome is similar with MPTP where effects are acute and insults to DA neuron are non-progressive. MPTP produces most but not all pathological hallmarks of PD.^68^ MPTP is a more favourable model than 6-OHDA because it is able to produce bilateral lesions, which is more relevant to PD since both hemispheres will have dopamine depletions consequently producing more PD-like symptoms.^62^ This bilateral lesion effect is achieved by systemic injections either i.p. or subcutaneously at higher doses in order to evoke desired PD symptoms while minimising toxic side effects of the intracranial injections. MPTP, when administered with probenecid, is more effective in producing similar pathological and clinical symptom in PD human as probenecid blocks MPTP clearance.^62^ This is, however, not to discount the significant roles of toxin-based animal models in the development of symptomatic therapies.^69^

We performed sensitivity analyses on different animal models used to assess if results changed. For rotarod analyses, one study used transgenic animal model, while the rest used toxin based. Of the eight studies that used toxin-based animal models, six studies used MPTP while two studies used 6-OHDA. We also tried excluding Chung *et al*^31^ due to cannabis introduction before and after toxin. We found that these sensitivity analyses did not significantly alter the overall results. This is similar to the pole test. Of the eight included studies, one study used transgenic mice, two studies used 6-OHDA toxin and the rest used MPTP toxin. The analyses did not significantly alter the results. For open field, of the six studies, two studies used transgenic animal models, two studies used MPTP and another two studies used 6-OHDA toxin. Due to the small number of studies included, we did not explore separating studies with these animal models. The aim of this review is to find if there is any effect of cannabinoids in PD animal models. Although there are significant differences between models, grouping all toxin-based animal models (with or without inclusion of the transgenic model) would make the most meaningful data for the review.

There are also differences with how human and animal models such as rodents metabolises environmental toxins, as well as differences in capacity in blood–brain barriers,^62^ hence interpretations from animal model experiments are to be taken with caution.

Most of the toxin-based studies in this review used male mice model. It is known that higher doses of MPTP can kill female mice.^70^ Traditional animal studies in PD use male mice. For studies that used both male and female mice, they commonly fail to provide comparison of outcomes between sex. This is a known limitation in translating positive animal studies to humans.^71^ Development of PD is twice higher for male than female. However, the latter has faster disease progression and higher mortality rate.^71^

Despite all the identified limitations, toxin-based animal models in PD are indispensable in understanding and finding therapeutic treatment. This review is not to investigate and present the histoclinical pathology of PD. This review aims to find any treatment effect of cannabis-based treatment in PD symptoms. Positive outcomes for both rotarod and pole tests did not significantly change even after analysing studies that only used MPTP-based animal models. A systematic review of cannabis derived phytocannabinoids (CDCs) in PD animal models demonstrated neuroprotective effects evidenced by increased dopamine and dopaminergic neurons levels.^72^ Cannabis was able to reduce losses of dopaminergic neurons^73 74^ and increase TH-positive neurons.^74–76^ The authors account these results from CDC’s ability to combat oxidative stress, reduce neuroinflammation and their antiapoptotic effects. These results may provide some support to why this review detected significant motor improvement of PD animal models.

We also noticed the variations on how the different outcomes were measured between studies. For rotarod, most of the studies evaluated rotarod using accelerating rotating speed between 4 and 40 rpm over 5 min^20 22 30 41 50 53^ while one study evaluated rotarod using accelerating speed up to 20 rpm over 20 min,^31^ 4–20 rpm over 3 min^51^ and another study 10–60 rpm over 5 min,^47^ taking the average of three trials for most studies. For pole test, the height of the pole ranges from 50 cm to 60 cm high, with diameter ranging between 0.8 cm and 1 cm between studies. Most of the studies took the average time to turn head and descend using the average of three trials, while one study measured time to descend to the floor. It is difficult to determine how these measurement variances affected the overall outcomes. However, because both tests have the same intent to measure motor performances and data were presented with similar measurement units, we decided to proceed to meta-analyses.

This improvement of motor function, however, was not replicated in the open field test with an SMD of 0.36 (95% CI −0.58 to 1.29; p=0.453). This could be due to the high heterogeneity between studies which included genetic-based and toxin-based PD models and the use of a variety of cannabinoids (two CBR agonists, three MAGL/FAAH inhibitor or endocannabinoid modulator) or more significantly, had variations in how the open field tests were undertaken in each included study. The duration of testing ranged from 5 min,^20 23 53^ 10 min,^34 59^ up to 15 min^54^ and the area of the test arena also differed between studies: 1 m × 1 m,^59^ 45 cm × 45 cm^20 23^ and unspecified space measurements.^34 53 54^ There are significant variabilities in published open field protocols in the literature,^65 66^ and there is a lack of reproducibility in studies when the same open field is carried out in different laboratories^63 64^ thereby making open field results challenging to compare and interpret.^66^

In some studies, multiple different doses of cannabinoid were used. For this meta-analysis, to ensure independence of study results, it was predetermined that only the highest dose was included in meta-analyses. This was based on an assumption of a dose-response effect if there was a detectable effect. It is possible that such an assumption is in error, though results from Shi *et al* and Chung *et al*, in which multiple doses were used, suggest that the highest dose did produce the largest effect (although lesser doses may have also produced an equivalent effect).

Another limitation of this study is the potential effects of publication bias and unclear and high risk of biases. Most studies did not specify details about baseline symptoms, randomisation or blinding processes. These are known limitations and are inherently problematic when conducting systematic reviews of preclinical studies. Included studies in this review also have variability in the toxin dosing, delivery method as well as in the treatment schedule and when it was commenced after the toxin (Table S9) on https://doi.org/10.6084/m9.figshare.19695004.v3).^18^ This would have likely influenced the extent of the disease observed in the animals and the subsequent behaviours. All these mentioned factors may have influenced the results of our meta-analyses. However, one potential benefit of the heterogeneity in the studies is that it might capture the different facets and diverse range of PD symptoms throughout disease progression. PD animal models are diverse and no one standard is used due to the difficulty in replicating the full myriad of PD symptoms in animal models.^77^

The results of this review do not guarantee successful translation clinically. In fact, the translation of preclinical results clinically has been elusive. Systematic reviews of medicinal cannabis in patients with PD showed subjective alleviation of motor and non-motor symptoms, however, the evidence is weak.^8 9^ More robust and symptom-specific RCTs are more required to further elucidate any cannabis effect. This review suggests that a study of cannabis on motor functions may provide the best clinical benefit for patients.

Overall, this systematic review and meta-analysis provides evidence of the benefit of cannabinoid treatment in PD animal models, which warrants further investigations. This review supports clinical trial of cannabis or cannabis-based treatments in humans with PD.

## Conclusion

This systematic review and meta-analysis provides evidence for the efficacy of cannabinoids in PD animal models. Meta-analysis of both rotarod and pole tests suggest an improvement in motor functions, and therefore, warrants further investigation of these outcomes clinically through cannabis clinical trials in patients with PD.

## Data Availability

Data are available in a public, open access repository. All data relevant to the study are included in the article. Data are available in a public, open access repository. All data relevant to the study are included in the article or uploaded as supplementary information via the URL provided. All supplementary figures and tables can be viewed and accessed in Figshare: https://doi.org/10.6084/m9.figshare.19695004.v3.^18^ Raw data can be viewed and access in Figshare: https://doi.org/10.6084/m9.figshare.21626600.v1.^78^ Also, Stata codes for meta-analyses can be viewed and access in Figshare: https://doi.org/10.6084/m9.figshare.21311169.v3.^79^ License: all are Creative Commons Attribution 4.0.
